# Beyond Small Molecules: Antibodies and Peptides for Fibroblast Activation Protein Targeting Radiopharmaceuticals

**DOI:** 10.3390/pharmaceutics16030345

**Published:** 2024-02-29

**Authors:** Xiaona Sun, Yuxuan Wu, Xingkai Wang, Xin Gao, Siqi Zhang, Zhicheng Sun, Ruping Liu, Kuan Hu

**Affiliations:** 1 School of Printing and Packaging Engineer, Beijing Institute of Graphic Communication, Beijing 102600, China; sxnxiaona1999@163.com (X.S.); wuyuxuan1111@outlook.com (Y.W.); sunzhicheng@bigc.edu.cn (Z.S.); 2 State Key Laboratory of Bioactive Substance and Function of Natural Medicines, Institute of Materia Medica, Chinese Academy of Medical Sciences & Peking Union Medical College, Beijing 100050, China; wangxingkai@imm.ac.cn (X.W.); gaoxin@imm.ac.cn (X.G.); zhangsiqi@imm.ac.cn (S.Z.)

**Keywords:** FAP, radiopharmaceuticals, PET imaging, theranostics, radionuclide therapy

## Abstract

Fibroblast activation protein (FAP) is a serine protease characterized by its high expression in cancer-associated fibroblasts (CAFs) and near absence in adult normal tissues and benign lesions. This unique expression pattern positions FAP as a prospective biomarker for targeted tumor radiodiagnosis and therapy. The advent of FAP-based radiotheranostics is anticipated to revolutionize cancer management. Among various types of FAP ligands, peptides and antibodies have shown advantages over small molecules, exemplifying prolonged tumor retention in human volunteers. Within its scope, this review summarizes the recent research progress of the FAP radiopharmaceuticals based on antibodies and peptides in tumor imaging and therapy. Additionally, it incorporates insights from recent studies, providing valuable perspectives on the clinical utility of FAP-targeted radiopharmaceuticals.

## 1. Introduction

Cancer is a heterogeneous disease that develops in an incredibly complex microenvironment [1,2,3,4,5,6]. This microenvironment encompasses not only cancerous cells but also vital constituents like the extracellular matrix [7,8,9,10], stromal cells [11,12,13], and immune cells [14,15,16], forming the tumor microenvironment (TME) [17,18,19,20]. The TME, an intricate milieu unique to tumors, exerts profound influences on tumor progression, immune evasion, metastasis, and therapeutic resistance [4,21,22,23,24,25,26].

Among the various types of fibroblasts implicated in cancer, cancer-associated fibroblasts (CAFs) stand out prominently [27,28,29,30,31,32,33,34]. These CAFs constitute a staggering 80% of all fibroblasts within the TME, assuming a pivotal role as oncogenic regulators with far-reaching effects on tumor cell proliferation, migration, extracellular matrix remodeling, and immunosuppression [35,36,37,38,39,40,41]. Originating from fibroblasts in normal tissues [42,43,44,45], these cells undergo a reversible transformation akin to myofibroblasts after injury, actively participating in wound healing [27,46,47,48,49]. Myofibroblasts transition from their initial presence in granulation tissue to become the predominant cell type in the proliferative phase, ultimately diminishing as the wound healing process concludes [50,51,52]. In the context of cancer, fibroblasts and other stromal cells orchestrate this transformation into CAFs by secreting transforming growth factors in the TME [27,46,53,54]. Notably, CAFs, in contrast to cancer cells, exhibit remarkable stability and resistance to drug resistance, underscoring their viability as a pivotal biological target for cancer diagnosis and therapy [55,56,57,58].

Identification of specific biomarkers on the surface of CAFs offers a strategic avenue for targeted radiological diagnostics and therapeutics [59,60,61,62,63,64]. Among these biomarkers, fibroblast activation protein (FAP) has gained widespread attention for its potential in CAF identification and targeting [65,66,67,68,69,70,71]. FAP, a member of the dipeptidyl peptidase 4 (DPP4) family, boasts a molecular weight of 170 kDa [72,73,74,75,76]. It assumes the guise of a type II transmembrane serine protease, typically existing as a homodimer [77,78,79]. Functionally, FAP exhibits dipeptidyl peptidase and endopeptidase activities [65,80,81,82], and its significance extends to normal embryonic development and tissue modeling [83,84,85]. Remarkably, FAP remains scarcely noticeable or entirely absent in normal adult tissues [86,87]. However, it undergoes marked upregulation during processes such as wound healing, atherosclerotic plaque formation, and fibrosis [88,89,90,91], and prominently features in over 90% of human epithelial carcinomas [72,80,92,93,94,95,96,97,98]. The consistent presence and overexpression of FAP in CAFs across numerous epithelial tumors, including colon, pancreatic, hepatic, and ovarian cancers, have paved the way for targeted FAP approaches in tumor imaging and therapy [99,100,101,102,103,104].

As an extensively explored target, especially in conjunction with positron emission tomography/computerized tomography (PET/CT) [105,106], various strategies including small molecules, peptides, and antibodies have emerged to harness FAP for tumor imaging and treatment [107,108,109,110,111]. Small molecule FAP inhibitors (FAPIs) have entered a new milestone since UAMC-1110 developed by Jansen et al., 2014 [112]. Numerous reviews on small molecule FAPIs have been summarized, such as the comprehensive review by Lan and Cai et al., 2022 [113]. While small molecule FAPIs have shown remarkable efficacy in tumor imaging, their therapeutic potential for tumors is limited by their short retention period in tumor tissues. In contrast, antibodies and peptides have longer half-lives in vivo and they can extend tumor retention time, thereby increasing tumor imaging signals. They also have better tissue permeability, which makes it easier for them to penetrate tumor tissue and generate stronger signals inside the tumor. In addition, antibodies and peptides are larger than small molecules, bind to targets more specifically, and generally have lower cytotoxicity and side effects. Therefore, the longer half-life, better tissue permeability, and lower toxicity of FAP-targeted peptides and antibodies relative to small molecule tracers make them a more effective choice for tumor imaging and treatment [114,115,116]. This focused review discusses the development and application of antibody- and peptide-based radiopharmaceuticals targeting FAP.

## 2. Antibody-Based Radiopharmaceuticals Targeting FAP

### 2.1. Iodine-131-Labeled Monoclonal Antibody F19

The discovery of FAP, a type II transmembrane serine protease, can be traced back to 1986 when it was initially identified as the F19 antigen during studies involving cultured fibroblasts and the monoclonal antibody (mAb) F19 [117,118,119]. Subsequently, in 1994, the surface antigen expressed by F19 cells was officially named FAP [86,120]. In 1990, Garin-Chesa et al. proposed that in the context of cancer, epithelial cancer, F19^+^ fibroblasts, colloquially referred to as FAP, emerged as a consistent molecular trait of the reactive stroma. The role of mAbF19 in their identification was pivotal [94]. Human FAP, discerned through mAbF19, became a prominent cell surface antigen [121]. Due to its abundant presence within the tumor mesenchyme, FAP can serve as a target for radionuclide antibody conjugates in cancer patients [121,122,123]. Various radionuclide-labeled antibodies, designed for FAP-targeted imaging and therapy, have been developed (Table 1).

In 1988, Old et al. conducted a comprehensive examination of six human cell surface glycoproteins, each defined by mAb, with the intention of characterizing the surface phenotype of cultivated mesenchymal cells [92]. Among these antibodies, mAbF19 effectively identified glycoproteins with molecular weights of 120,000 and 95,000, expressed on cultivated fibroblasts and a proportion of sarcoma cell lines, respectively. This discovery marked mAbF19 as a superior antibody for these purposes compared with other candidates, such as mAbF24, G171, G253, and K117. Another antibody, S5, exhibited expression patterns similar to mAbF19 but had limited in vivo expression [92]. It became evident that the fibroblasts surrounding tumor cells offer effective targets for cancer immunolocalization or immunotherapy, owing to their recognition by mAbF19 [129,130,131].

**Figure 1:**
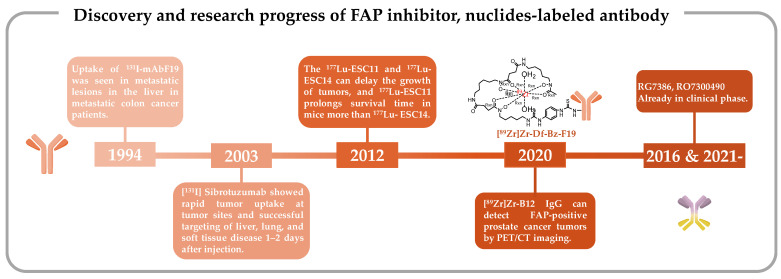
Discovery and research progress of nuclide-tagged FAP-targeting antibodies [126,127,128,132,133,134].

The pioneering clinical study involving FAP-targeting radiopharmaceuticals utilized ^131^I-labeled mAbF19 for tumor imaging in patients with liver metastases from colon cancer [132]. Welt et al. conducted this study in 1994, wherein 17 patients scheduled for resection of localized metastases or regional chemotherapy received intravenous administration of ^131^I-mAbF19. Imaging results from this series of studies revealed that the tumor-to-normal tissue ratio reached its peak after 3–5 days of administration, enabling the visualization of lesions as small as 1 cm in diameter. Notably, colon cancer studies demonstrated specific localization of tumors and metastatic lesions. However, SPECT/CT results revealed slow kidney clearance, necessitating 3–5 days to achieve optimal imaging outcomes. This delayed renal clearance has implications for the imaging capabilities of ^131^I-mAbF19 (Figure 1) [132].

### 2.2. Iodine-131-Labeled Sibrotuzumab

mAbF19 stands out for its FAP-specific targeting capabilities, and scientists have endeavored to enhance its imaging potential as a radiopharmaceutical [135]. A study by Welt et al. established the positive expression of FAP in all 17 patients studied, and SPECT/CT imaging effectively facilitated precise tumor identification in humans [132]. This encouraged further investigation into the pharmacokinetic (PK) of sibrotuzumab, a humanized version of the murine anti-FAP mAbF19. A phase I and II clinical trial (NCT02198274) involving sibrotuzumab sought to assess its PK without radiolabeling in patients with FAP-positive malignancies, particularly advanced metastatic colorectal cancer patients. However, among the 17 patients enrolled who had undergone rigorous pretreatment, only two exhibited stable disease status, a count insufficient to meet the phase II trial criteria, which typically necessitate four patients in a stable condition or at least one patient in complete or partial remission. Consequently, the trial did not progress beyond phase II [136]. The PK study unveiled pertinent findings, including a mean clearance rate of 39.8 ± 19.8 mL/h and a terminal half-life of 5.3 ± 2.3 days for sibrotuzumab. Comparatively, mAbF19 displayed a mean clearance rate of 109 mL/h and a terminal half-life of 38 h. Sibutuzumab’s lower clearance rate and significantly protracted half-life rendered it a more amenable candidate for radioimmunotherapy. However, this study underscored the inadequacy of unlabeled sibrotuzumab in the treatment of advanced solid tumors, instigating investigations into sibrotuzumab radioisotope conjugates [136].

One such avenue of exploration encompassed a clinical phase I dose-escalation study of sibrotuzumab in patients with colorectal or non-small cell lung cancer (NCT02209727). This study, conducted in parallel with the aforementioned phase I/II trials, sought to evaluate biodistribution, PKs, immunogenicity, and safety profiles by incrementally administering sibrotuzumab intravenously to 26 patients. In tandem, two antecedent phase I studies employed ^131^I-mAbF19, with a focus on patients with hepatic metastases from colorectal cancer or soft tissue sarcoma [137]. These investigations delved into the PK parameters of the therapeutic mouse monoclonal antibody ^131^I-mAbF19, elucidating principles of selective tumor accumulation and stromal targeting in tumors through biodistribution imaging studies and biopsy analyses. These pivotal insights provided the foundation for the inaugural human clinical evaluation of sibrotuzumab.

In this human trial, patients received 8–10 mCi of ^131^I-labeled sibrotuzumab, administered concomitantly at weeks 1, 5, and 9 in a 12-week dosing cycle, with a focus on PK assessments. The ensuing analysis divulged that the mean clearance rate of ^131^I-sibrotuzumab amounted to 41.9 ± 16 mL/h, accompanied by a half-life of 4.9 days. Following a single cycle of ^131^I-sibrotuzumab treatment, two out of the 26 patients manifested stable disease conditions. The relatively abbreviated half-life of sibrotuzumab held promise for radioimmunotherapy. Regrettably, the trial did not yield definitive efficacy results for sibrotuzumab, prompting the discontinuation of further clinical development (Figure 1) [133]. Furthermore, it is noteworthy that despite considerations of alternative targets, the commercial viability of ^131^I-sibrotuzumab waned, ultimately leading to its discontinuation in 2014 [125].

### 2.3. ^177^Lu-ESC11/ESC14

The clinical application of ^131^I-mAbF19 and its humanized derivative, sibrotuzumab, has been hindered by their prolonged blood clearance and suboptimal therapeutic efficacy. To enhance the therapeutic potential of FAP-targeted antibodies, it is imperative to develop antibodies with enhanced attributes and utilize radiolabeling with more suitable radionuclides [138,139,140,141]. In addressing these considerations, Fischer et al. introduced a significant advancement with the development of ^177^Lu-ESC11 and ^177^Lu-ESC14 [126].

From the perspective of antibody discovery, ESC11 and ESC14, two antibodies noted for their selective accumulation within xenografted FAP-positive human melanoma and their capacity to impede tumor growth in vivo, were identified in the human FAP antibody library using the phage display technique. Subsequently, these antibodies underwent a transformation into IgG1 antibodies [126]. The phage display technique is a new technique predicated on specific affinity interactions, enabling the identification of proteins or peptides that exhibit particular binding properties. This technique is especially adept at discovering antibodies targeting challenging and intriguing molecules, making it a cornerstone in the quest for antibodies with precise attributes [142,143,144,145,146]. It enjoys widespread utilization in the quest for human antibody fragments boasting specific binding activity [146,147,148]. This technique facilitates the evolution and optimization of FAP-targeting antibodies, culminating in the selection of antibodies exhibiting robust affinity, rapid internalization, and propensity for tumor accumulation. Following their identification, ESC11 and ESC14 obtained by screening were labeled with lutetium-177 (^177^Lu), leading to a comprehensive investigation into their in vivo targeting capabilities. The outcomes of these investigations substantiated that both modified mAbs can selectively bind to human and mouse FAP, featuring affinities of 10 nM and 210 nM, respectively (Figure 1) [126].

From a radionuclide perspective, radiolabeling with iodine-131 is relatively straightforward for mAbs [149]. However, in the context of radioimmunotherapy, iodine-131 proves to be suboptimal due to its propensity for facile release from tumor sites after internalization of mAbs within cells [150,151]. A related drawback lies in the emission of high-energy (364 keV) γ-photons, accounting for a substantial 82% of its radiation output, thus posing concerns for radiation safety [105,152]. In stark contrast, the radioactive lanthanide ^177^Lu presents a more favorable profile, with a shorter emission range of 2 mm as opposed to iodine-131’s 3 mm. Additionally, ^177^Lu exhibits markedly improved physical radiation characteristics, featuring the emission of 208 keV γ-photons at a much lower abundance (11%) [126]. As a result of these considerations, Fischer et al. chose ^177^Lu for antibody labeling. This decision culminated in the successful radiolabeling of ESC11 and ESC14 through their conjugation with CHX-A″-DTPA.

These radiolabeled antibodies were subsequently administered to mice harboring SK-Mel-187 and SK-Mel-16 xenograft tumors to assess their tumor uptake. In this investigation, ^177^Lu-CHX-A″-DTPA-vF19 and ^177^Lu-CHX-A″-DTPA-A33 were included as control groups. Notably, SPECT/CT imaging conducted 72 h post-injection revealed a higher specific uptake of ^177^Lu-ESC11 in SK-MEL-187 tumors, whereas SK-MEL-16 xenografts exhibited lower uptake compared with the control group. This study, involving a comparative analysis of the in vivo targeting attributes of human–mouse chimeric antibodies, established that in mouse models characterized by higher levels of antigen expression, the cumulative tumor uptake of the nuclide-labeled antibody could reach levels corresponding to 50% of the administered dose per gram. Conclusive in vivo experiments in mice further corroborated that the ratio of tumor-to-organ uptake pertaining to ^177^Lu-labeled FAP mAbs ESC11 and ESC14 surpassed that of their first-generation radionuclide-labeled FAP-targeted antibodies [126]. The novel antibodies, ESC11 and ESC14, exhibited efficient internalization into FAP-expressing cells, thereby manifesting highly satisfactory in vivo targeting capabilities.

### 2.4. ^89^Zr-Labeled F19 and B12 IgG

Clinical investigations involving first-generation FAP-targeting antibodies have provided novel insights by demonstrating the feasibility of modifying FAP-specific cancer targeting through the conjugation of toxins or chelators with FAP-specific antibodies [153,154]. As an illustrative example, Pandya et al. prepared the radiopharmaceutical antibody conjugate [^89^Zr]Zr-Df-Bz-F19 mAb for PET imaging by employing the bifunctional chelator Df-Bz-NCS to securely bind zirconium-89 (^89^Zr) (Figure 1) [127].

^89^Zr, a radionuclide, emits β+ particles at 902 keV with an abundance of 23% and possesses a half-life of 78.4 h [105,155]. Its attributes, characterized by high-resolution imaging, specific tissue binding, and strong signal contrast, render it a promising candidate for PET imaging applications [156,157]. Nonetheless, certain challenges persist, primarily the susceptibility to covalent bond breakage between the chelator and the protein, which can compromise stability [158]. To mitigate this concern, comprehensive in vitro characterization of [^89^Zr]Zr-Df-Bz-F19 mAb was conducted. The radiolabel displayed a remarkable radioactive purity exceeding 99.5% upon synthesis completion and retained its purity at levels greater than 99.1% in human serum after 7 days of incubation at 36–37 °C. These findings affirm the robust stability of nucleoporin labeling and underscore the ability of [^89^Zr]Zr-Df-Bz-F19 to maintain its structural integrity following in vivo administration.

In vivo experiments in U87MG tumor-bearing mice unveiled the rapid clearance of [^89^Zr]Zr-Df-Bz-F19 from the bloodstream, with 51% of the radioactive drug eliminated from the blood within the timeframe spanning 2 to 72 h. Notably, ^89^Zr was observed to accumulate in bone tissues. Furthermore, the study revealed an increase in the average tumor-to-kidney (T/K) and tumor-to-blood uptake ratios over time, specifically at 2, 24, 48, and 72 h. After 72 h, the metabolism of the ^89^Zr labeled through bifunctional chelation of F19 became apparent. The exceptional radioactive purity, in vivo stability, and favorable tumor-to-tissue uptake ratio collectively position [^89^Zr]Zr-Df-Bz-F19 as a promising candidate for PET/CT imaging [127].

FAP expression is documented in multiple solid cancers, yet limited knowledge exists regarding its prevalence in metastatic castration-resistant prostate cancer (mCRPC) [159,160,161,162]. The evolving landscape of precision treatment strategies for prostate cancer was highlighted in the European Society of Medical Oncology 2022 report [163,164]. Advancements in CRPC therapies hinge on accurate imaging modalities, lesion visualization, disease staging, and informed therapeutic decision-making [105]. These findings underscore the ongoing demand for selective and sensitive imaging probes applicable to mCRPC patients.

To address the specific context of mCRPC, Hallie et al. employed a humanized antibody, initially identified by phage display, and labeled it with ^89^Zr. This antibody serves as a FAP-expressing tumor-selective imaging probe for PET/CT imaging in a preclinical prostate cancer xenograft model [128]. The investigative process commenced with genomic and immunohistochemistry assessments to determine the expression of FAP in prostate cancer [128,165,166]. Specifically, FAP localization in tissues and cells was determined via antigen–antibody binding reactions. Genomic analysis was predicated on RNA sequencing derived from primary prostate cancer patient samples or mCRPC bone and soft tissue tumor biopsies [167,168]. Concurrently, immunohistochemistry analyses were performed on cancer samples from a biological materials repository [128]. These comprehensive genomic and immunohistochemistry investigations unequivocally demonstrated FAP expression across transgenomic subtypes of metastatic disease and metastatic sites. Importantly, this affirmed FAP’s utility as a target imaging and therapeutic intervention within the prostate cancer tumor microenvironment [128,169].

Further validation entailed assessing the designed probe’s specificity for FAP-expressing tumors using near-infrared (NIR) dye-labeled B12 IgG. The outcomes affirmed that B12 IgG effectively and specifically detects cells expressing FAP by NIR fluorescence imaging [170]. This critical step established the foundation for the prospective development of B12 IgG as a contrast agent for PET imaging.

Subsequent PET/CT imaging evaluations were conducted in a mouse model established through the subcutaneous injection of CWR-R1FAP cells. Notably, [^89^Zr]Zr-B12 IgG demonstrated significantly enhanced tumor uptake in FAP-positive cells in mice bearing CWR-R1FAP relative to the control group injected with [^89^Zr]Zr-IC IgG. This heightened tumor accumulation is attributed to improved permeability and retention efficiency, leading to a sustained presence of the [^89^Zr]Zr-IC IgG probe within the tumor tissue, unlike the control probe, which exhibited rapid clearance and near-invisibility at 72 h. Compared with that in the control group, there was a heightened accumulation of [^89^Zr]Zr-B12 IgG in FAP-positive tumor cells (Figure 1) [128].

In a critical extension of this work, mice were subcutaneously injected with hPrCSC-44 (an immortalized human prostate cancer stromal cell line) in combination with DU145 (a FAP-null prostate cancer cell line) to establish a xenograft mouse model. Subsequent administration of [^89^Zr]Zr-B12 IgG or control [^89^Zr]Zr-IC IgG in mice bearing subcutaneous hPrCSC-44/DU145 xenografts, followed by serial imaging was performed at 24, 48, 72, 96, 120, and 144 h post-injection, allowed for comprehensive PET imaging evaluation. The results showed maximum tumor uptake of [^89^Zr]Zr-B12 IgG at 24 h, which surpassed control levels by 4–5 times. Additionally, pronounced [^89^Zr]Zr-B12 IgG uptake in the liver was observed. Over time, tumor and liver uptake exhibited parallel reductions, with diminished tumor uptake evident at 144 h after injection [128].

[^89^Zr]Zr-B12 IgG has the potential for noninvasive PET/CT imaging applications in mCRPC, serving as a specific imaging probe for FAP-expressing tumors. Notably, B12 IgG can be internalized by FAP-expressing cells, as evidenced by its robust tumor accumulation. Furthermore, it demonstrates an adeptness for preserving imaging signals and holds promise for immunoconjugate-based therapies. Importantly, B12 IgG facilitates facile nuclide exchange through chelator conjugation, rendering it a promising candidate for radioimmunotherapy applications [128].

### 2.5. Bispecific Antibodies

In the realm of tumor-targeting antibodies, an exciting development involves Hoffmann LaRoche’s bispecific antibodies RG7386 (FAP-DR5) and RO7300490 (FAP-CD40) (Figure 1) [134,171,172]. Currently in phase I clinical trials (NCT02558140, NCT04857138) [173,174], these antibodies represent a novel approach. One of these antibodies is engineered to target FAP for precise localization, while the other is designed to interact with molecules influencing tumor apoptosis or necrosis. These bispecific antibodies work in tandem, obstructing different signaling pathways simultaneously. Compared with monoclonal antibodies, bispecific antibodies possess two specific antigen-binding sites, which endow them with stronger specificity. Consequently, they precisely target tumor cells, minimize off-target toxicity, and can orchestrate immune cell-mediated tumor eradication through dual-target signal blockade [175,176,177,178,179,180,181,182]. This special structure confers a unique advantage to bispecific antibodies in the field of tumor therapy. Notably, LaRoche’s bispecific antibody, approved in 2017, achieved a revenue of USD 3.5 billion in 2021, establishing biphasic drugs as a prominent topic of interest following the success of PD-1 inhibitors.

The concept of bispecific antibodies also opens up new possibilities for radionuclide labeling. While no specific studies have explored the radionuclide labeling of bispecific antibodies targeting FAP, research into combination therapies involving radionuclides and bispecific antibodies targeting other antigens has been conducted [183,184]. For instance, Morris’ team combined radionuclides with bispecific antibodies (anti-CTLA-4 and anti-PD-L1). In a mouse model, this approach resulted in complete and enduring tumor remission, outperforming combinations involving monoclonal antibodies [185]. The potential to combine or label FAP-targeted bispecific antibodies is a promising avenue that introduces a fresh dimension to radiotherapy nuclide markers for FAP-targeted tumor therapy. Such innovations hold significant potential for advancing clinical FAP-targeted tumor therapy.

## 3. Peptide-Based FAPIs

### 3.1. Peptide-Based FAP Radiopharmaceuticals

While radiolabeled antibodies against highly expressed FAPs on fibroblasts exhibit high affinity and specificity, their clinical utility in molecular imaging is hindered by prolonged circulation in the bloodstream and limited tumor penetration potential [186]. In contrast, small peptides typically exhibit favorable PK profiles characterized by rapid clearance from tissues and efficient tumor penetration [105,187,188,189,190,191,192]. As such, peptide-based radiopharmaceuticals have emerged as the most promising candidates for molecular imaging in the clinical setting [193,194,195,196,197,198,199,200,201,202,203,204,205,206]. Cyclic peptides, in particular, offer enhanced structural stability and resistance to enzymatic degradation compared with linear peptides, greatly enhancing their efficacy in radiological diagnosis and therapy [207,208,209,210,211,212,213,214,215]. FAP-targeted cyclic peptides have shown encouraging outcomes across numerous clinical trials.

For instance, 3B Pharmaceuticals introduced 263 different FAP-targeted peptide structures and identified peptides suitable for radiolabeling (Table 2). Notably, 3BP-3320, 3BP-3321, 3BP-3407, and 3BP-3554 exhibited exceptional radioactive purity. The selected polypeptides were labeled with indium-111 and subsequently injected into mice to evaluate the tissue-specific radioactive uptake. The findings highlighted that ^111^In-3BP-3554 displayed the highest tumor/tissue radioactivity uptake rate. Consequently, this tracer underwent in-depth in vivo PK evaluation in mice. Representative SPECT/CT imaging was performed three hours after injection of ^111^In-3BP-3554 in four different mouse sarcoma tumor models, consistently revealing significant radioactive uptake in all tumor types. Dose–response evaluations of ^177^Lu-3BP-3554 were further conducted in the Sarc4809 model. Compared with ^nat^Lu-3BP-3554, ^177^Lu-3BP-3554 demonstrated marked inhibitory effects on tumors, with increasing doses amplifying this effect [216]. On 21 September 2019, Clovis Oncology secured an upfront payment of USD 12 million for exclusive rights to FAP-targeted radiolabeled peptidomimetics (FAP-2286) developed by 3B Pharmaceuticals, with the exception of Europe [217].

### 3.2. Structure of the Selective and Specific Peptide FAPI

**Figure 2:**
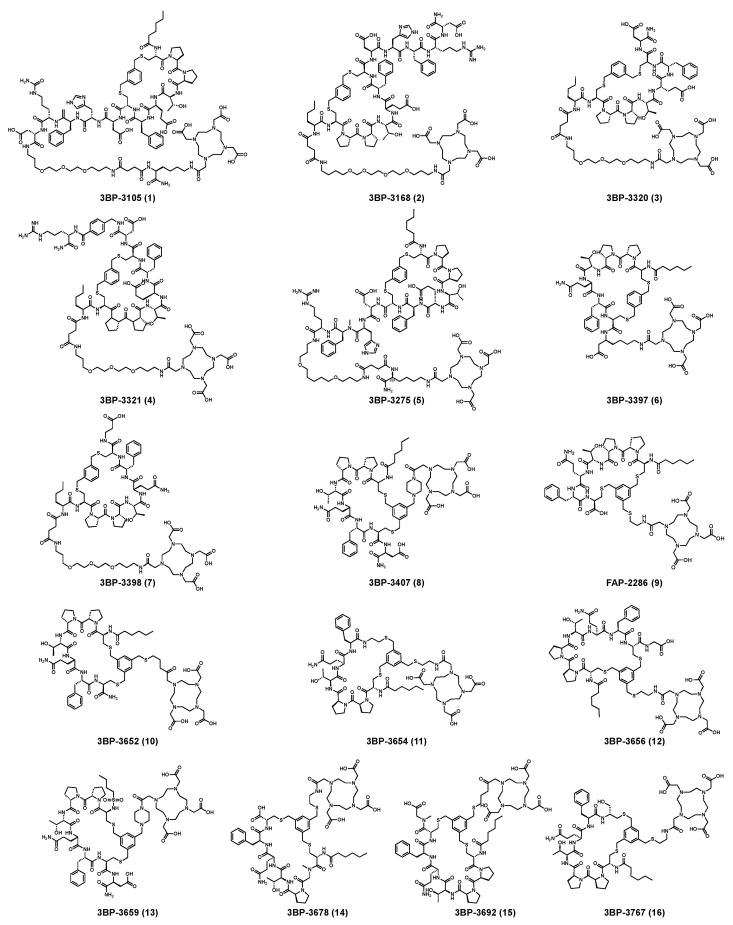
Cyclic peptide compounds of 3B Pharmaceuticals [216].

In their patent, 3B Pharmaceuticals has cataloged a range of peptide structures amenable to radiolabeling with radionuclides (Table 3 and Figure 2). Among these structures, 3BP-3554 emerged as a potential clinical candidate and was renamed FAP-2286.

Distinguishing features between FAP-2286 and other peptides within the same series manifest chiefly in the following facets. Firstly, the placement of the chelator dramatically affects both the radiopurity of the nuclide-labeled compound and its tumor uptake rate. When the chelator is linked to the tMeBn structure, which is instrumental in cyclization, it enhances radiopurity and results in a higher tumor uptake rate compared to compounds where the chelator is linked to the polypeptide chain. Moreover, different modifications of cyclic peptides can affect the compound’s specific affinity and polarity. For instance, altering only the cysteine substituent at the C-terminus of the cyclic peptide leads to corresponding changes in specific affinity. Moreover, extending the length of the chain segment impacts the compound’s polarity. In the absence of substituents, longer chain segments decrease molecule polarity, thereby diminishing polarity [216,218].

FAP-2286 demonstrates highly effective and specific binding to FAP of human and mouse origin, along with a pronounced inhibitory effect. The equilibrium dissociation constant (KD) values were 1.1 and 4.7 nM, and the half maximal inhibitory concentration (IC_50_) values were 3.2 and 22.1 nM for human and mouse FAP, respectively. The metal complexes of FAP-2286 exhibited potent binding to human and mouse FAP, featuring KD values of 0.2–1.4 nM and 1.9–7.7 nM, respectively. Importantly, these metal complexes did not compromise the inhibitory activity against FAP, affirming their exceptional FAP specificity. Mean IC_50_ values for FAP from human and murine sources were determined as 1.3–2.2 nM and 8.4–16.3 nM, respectively [216,219].

Furthermore, FAP-2286 has favorable stability, high radioactive purity after radionuclide labeling, and outstanding specific affinity. Consequently, Clovis Oncology and 3B Pharmaceuticals have embarked on collaborative initiatives to conduct preclinical imaging and therapeutic evaluation of FAP-2286. Concurrently, Clovis Oncology has initiated plans for clinical trials involving FAP-2286 [220,221].

### 3.3. ^68^Ga-FAP-2286 and ^111^In-FAP-2286 for Nuclear Imaging

Gallium-68-labeled FAP-2286 demonstrates remarkable efficacy in tumor imaging. Consequently, 3B Pharmaceuticals directed their attention toward FAP-2286 and its chelation with natural nonradioactive metals (^nat^Ga-FAP-2286, ^nat^Lu-FAP-2286, and ^nat^In-FAP-2286), and proceeded to evaluate three radiotracers for their in vitro affinity and selectivity [216].

The excellent affinity, precision in targeting, and inherent stability of FAP-2286 and its complexes have been conclusively established, facilitating the coordination of gallium-68 and indium-111 radionuclides with FAP-2286 for use as PET or SPECT imaging agents, respectively [216]. In vivo SPECT imaging employing ^111^In-FAP-2286 in HEK-FAP tumor-bearing mice showcased stable accumulation within tumor tissues, accompanied by minimal uptake in non-tumor regions. Notably, the kidney displayed the highest nontargeted uptake, albeit the T/K ratio gradually declined over time, reaching its zenith at 48 h after treatment [216,219].

Compared with the small molecule FAPI series, cyclic peptide FAPIs manifested superior biological properties [222], including stronger receptor selectivity and binding affinity and longer tumor retention. Remarkably, ^68^Ga-FAP-2286 exhibited the same rapid renal clearance as ^68^Ga-FAPI-46, with no noticeable differences in tumor distribution between the two tracers [220]. Chen et al. performed a comparative uptake analysis of ^68^Ga-FAPI-46 and ^68^Ga-FAP-2286 in cancer patients to delineate the in vivo distribution patterns of different inhibitors [223]. ^68^Ga-FAP-2286 exhibited lower physiological uptake in muscles, salivary glands, thyroid, and pancreas than ^68^Ga-FAPI-46. Conversely, ^68^Ga-FAP-2286 displayed heightened uptake in the heart, kidneys, and liver relative to ^68^Ga-FAPI-46. Despite ^68^Ga-FAPI-46 undergoing clinical imaging investigations for diverse tumor models, its rapid blood clearance and limited tumor retention pose substantial limitations for diagnostic and therapeutic applications [224]. In preclinical studies, FAP-2286 demonstrated longer tumor retention and stronger antitumor activity over time than FAPI-46, maintaining consistent tumor uptake at 3 h post-injection. Key advantages inherent to FAP-2286 encompass elevated affinity for FAP binding, improved tumor accumulation, and prolonged tumor retention [223].

With its introduction into clinical practice, Clovis Oncology embarked on the radiolabeling of FAP-2286 with gallium-68 for clinical tumor imaging. In a phase I clinical trial (NCT04939610), ^68^Ga-FAP-2286 was used as a contrast agent for pretreatment PET scans in 30 patients with solid tumors. This approach will persist as a guide for pretreatment imaging and posttreatment evaluation in an ensuing phase II trial. Furthermore, Clovis Oncology has initiated a clinical evaluation of ^68^Ga-FAP-2286 (NCT04621435), which, as of June 2022, encompassed 48 patients with cancers of the breast, bladder, prostate, colon, head/neck, pancreas, sarcoma, cholangiocarcinoma, and lung [225]. Patients were subjected to ^68^Ga-FAP-2286 administration and imaged 64 ± 7 min after injection. Notably, cholangiocarcinoma exhibited the highest tumor uptake. PET imaging conducted on a 72-year-old patient with cholangiocarcinoma revealed peak tumor uptake at 120 min, with the tumor-to-background ratio progressively augmenting from 30 to 120 min. ^68^Ga-FAP-2286 PET emerges as a pivotal tool for staging patients across cancer types characterized by robust tumor uptake, renal metabolism, and negligible renal accumulation. Its application prospects are indeed promising [225].

### 3.4. ^177^Lu-FAP-2286 for Radionuclide Therapy

FAP-2286 can be radiolabeled with the radionuclide ^177^Lu for the treatment of clinical solid tumors. Prior to clinical trials, the therapeutic efficacy and specificity of this approach were evaluated in murine models. Notably, the administration of ^177^Lu-FAP-2286 to HEK293-FAP tumor-bearing mice and FAP-expressing xenografts mice from sarcoma patients did not result in significant weight loss. Furthermore, the tumor retention of ^177^Lu-FAP-2286 exceeded that of ^177^Lu-FAPI-46. Over the 24–72 h timeframe, the T/K ratio for ^177^Lu-FAP-2286 consistently increased, in contrast to the T/K ratio for ^177^Lu-FAPI-46, which peaked at 24 h. Importantly, ^177^Lu-FAP-2286 exhibited commendable properties as an active targeting agent, characterized by potent and specific FAP binding, resulting in high tumor uptake, accumulation, and demonstrable therapeutic effects [220].

Furthermore, clinical trials of FAP-2286 revealed prolonged tumor retention and superior tumor suppression compared with FAPI-46 [220]. Clovis Oncology initiated the LuMIERE phase I and II clinical trials of FAP-2286 (NCT04939610), enrolling patients with advanced solid tumors. Phase I focused on evaluating the safety and tolerability of ^177^Lu-FAP-2286, while phase II aims to determine the recommended dose of ^177^Lu-FAP-2286 and assess the objective response rate in patients. Phase I entails fixed-dose intravenous administration of ^177^Lu-FAP-2286 at six-week intervals, up to a maximum of six doses for patients exhibiting positive uptake of ^68^Ga-FAP-2286. The dose-escalation range, guided by Bayesian optimal interval design, spans between 3.7 and 9.25 GBq, encompassing four distinct doses (3.70 GBq/100 mCi, 5.55 GBq/150 mCi, 7.40 GBq/200 mCi, 9.25 GBq/250 mCi). Phase II involves intravenous administration of the recommended dose of FAP-2286 to a cohort of up to 40 patients with advanced solid tumors. As of October 2022, ^177^Lu-FAP-2286 is being examined with a 7.40 GBq metering regimen, with the LuMIERE study projected to conclude by 1 June 2026 [226].

The LuMIERE study has yielded noteworthy findings. In the phase I dose study, 11 patients underwent ^68^Ga-FAP-2286 imaging and received treatment with ^177^Lu-FAP-2286 across cohorts. Among these patients, three individuals with peritoneal pseudomucinous tumors or colorectal cancer received 3.70 GBq, while six patients with different solid tumors received 5.55 GBq, and two patients were administered 7.40 GBq of ^177^Lu-FAP-2286. Encouraging treatment outcomes were observed, with eight patients discontinuing treatment, and one patient, after completing six doses of 3.7 GBq ^177^Lu-FAP-2286 for more than 12 months, demonstrated stable disease without the requirement for subsequent anticancer interventions. Another with gallbladder adenocarcinoma, from the 5.55 GBq cohort, exhibited stable disease upon four doses. Notably, ^177^Lu-FAP-2286 exhibited a manageable safety profile with some preliminary evidence of antitumor activity [225].

**Figure 3:**
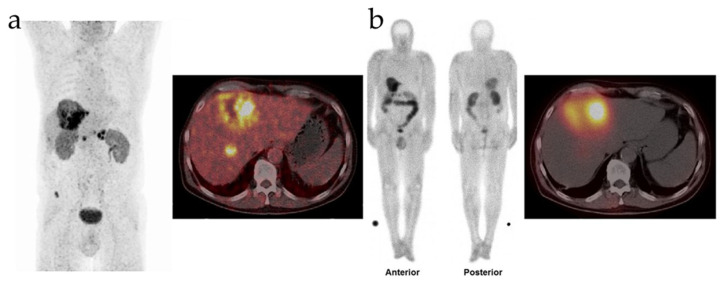
Therapeutic comparison of ^177^Lu-labeled targets. (**a**) PET images and a transverse PET/CT image captured with ^68^Ga-FAP-2286 before treatment for the patients. (**b**) Anterior and posterior views and transverse SPECT/CT images at 48 h after ^177^Lu-FAP-2286 injection. Figures from Baum et al. [221].

Prof. Richard P. Baum released findings pertaining to the biodistribution and preliminary dosimetry from the first human trial of FAP-2286 in March 2022. Intravenous injection of ^177^Lu-FAP-2286, completed within 5–10 min, was performed on 11 patients with advanced adenocarcinoma, all of whom had undergone ^68^Ga-FAP-2286 or ^68^Ga-FAPI-04 PET/CT imaging. SPECT/CT imaging after treatment illustrated a significant uptake of ^177^Lu-FAP-2286 within the tumor lesions. Notably, Patient 4 with pancreatic cancer and liver, peripancreatic lymph node, and bone metastases, underwent PET/CT imaging before the intravenous injection of ^177^Lu-FAP-2286. The patient’s anterior and posterior SPECT/CT images 48 h post-injection illustrated a significant liver uptake of ^177^Lu-FAP-2286 (Figure 3a). Patient 6 with breast cancer, characterized by diffuse FAP-positive bone and bone marrow metastases and lymph node metastases in ^68^Ga-FAP-2286 PET/CT imaging, displayed regression of bone and bone marrow lesions within 10 days (Figure 3b) [221].

Coincidentally, in November 2022, Rao et al. administered [^177^Lu]Lu-FAP-2286 to a patient with systemic metastases from squamous cell carcinoma of the right lung [227]. This intervention was followed by meticulous pre- and post-treatment assessments through [^68^Ga]Ga-FAP-2286 PET/CT imaging. The MIP image revealed systemic metastases from the patient’s squamous cell carcinoma of the right lung, supraclavicular lymph nodes, irregular thickening on the right side, hypodense hepatic nodules, and osteolytic lesions in the left scapula. Following nine weeks of injection with [^177^Lu]Lu-FAP-2286 at a dose of 7.0 GBq, [^68^Ga]Ga-FAP-2286 PET/CT scans exhibited a reduction in the affected regions within the MIP images [227]. The above two investigations have provided clinical substantiation of FAP-2286’s efficacy in treating FAP-positive tumors, thereby implying its potential utility in addressing a spectrum of advanced primary tumors and metastases. However, both studies have certain limitations. Firstly, the patient cohort in these studies was relatively small and displayed heterogeneity. Secondly, the administration of ^177^Lu-FAP-2286 was performed as the last-line treatment, precluding a dose-escalation regimen. Finally, the assessment of the safety and efficacy of ^177^Lu-FAP-2286 hinged primarily on observational data. Therefore, it is imperative that comprehensive clinical trials are undertaken to thoroughly investigate the PK, safety profile, dosimetry, and therapeutic efficacy of ^177^Lu-FAP-2286 in patients with advanced solid tumors [105].

^177^Lu-FAP-2286 has demonstrated substantial therapeutic promise, with ^177^Lu serving as the radiolabeled nuclide pivotal in this therapeutic paradigm. Various ^177^Lu-labeled drugs targeting diverse molecular entities have received regulatory approval from the U.S. Food and Drug Administration (FDA) [228]. For example, ^177^Lu-PSMA-617, designated for treating male patients with PSMA-positive mCRPC, exhibited a noteworthy effective systemic half-life of 40 h and entailed a mean absorbed red marrow dose of 0.03 GBq [228,229]. Similarly, ^177^Lu-DOTATATE, tailored for managing neuroendocrine tumors, boasted a prolonged effective systemic half-life of 55 h, accompanied by a mean absorbed red marrow dose of 0.04 GBq [228,230]. Comparatively, ^177^Lu-FAP-2286 manifested an effective systemic half-life of 35 h, coupled with a mean absorbed red marrow dose of 0.05 GBq. Importantly, the effective absorbed dose of ^177^Lu-FAP-2286 aligned closely with that of ^177^Lu-DOTATATE and ^177^Lu-PSMA-617, underscoring its compatibility for therapeutic application across a broad spectrum of malignancies. In addition, peptide-targeted radionuclide therapy with ^177^Lu-FAP-2286 relieves pain symptoms in invasive adenocarcinoma cases. Encouragingly, ^177^Lu-FAP-2286 also showed considerable potential in tumor remission and inhibition, shedding new light on FAP-targeted peptide-based radionuclide therapy. In the quest for improved efficacy, modifying the radionuclide payload holds promise. For instance, while ^177^Lu emits gamma (γ) rays and β-particles and alternative radionuclides such as actinium-225 (^225^Ac) and radium-223 (^223^Re) emit α-rays capable of inducing DNA double-strand breaks, rendering them potentially more lethal than β-particles [105]. Presently, multiple enterprises are engaged in clinical investigations into FAP-2286, further underscoring its burgeoning status as an emerging therapeutic agent poised for future market introduction.

## 4. Conclusions

Since the seminal discovery by Garin-Chesa et al., 1990, which revealed the high expression of FAP in most epithelial tumor cells [94], significant strides have been made in the realm of FAP-targeted therapeutics encompassing antibodies and peptides. Radiolabeled antibodies have long stood as exemplars of high targeting affinity and specificity. Their high molecular weight translates to protracted circulation in the bloodstream and tumor retention time, but correspondingly sluggish clearance [231,232,233]. Peptide drugs are characterized by relatively low molecular weight and can offer better blood circulation and tumor retention. Peptides, owing to their potential for heightened receptor selectivity, binding affinity, and tumor retention, may outstrip other FAP inhibitors in the realms of diagnostic and therapeutic efficacy [234,235,236,237,238,239]. At present, FAP tracers have great prospects in clinical application. Li et al. reported the efficacy evaluation of a 73-year-old male patient with recurrent bladder tumor after receiving ^177^Lu-FAP-2286 [240]. PET/CT results showed a decrease in the number and degree of uptake at the lesion sites. In addition, there are many clinical trials underway for FAP-2286, which involve solid tumors such as breast, pancreas, sarcoma, prostate cancer (NCT04621435) [241], and pathologic fibrosis such as idiopathic pulmonary fibrosis, cirrhosis, and cardiac fibrosis (NCT05180162) [242]. In conclusion, FAP has emerged as a prominent target in the pursuit of tumor-specific interventions, demonstrating promising outcomes in tumor imaging and therapy research. Nonetheless, the present study’s limitations, including a relatively small sample size and abbreviated follow-up duration, underscore the imperative for extensive future investigations and clinical trials to explore the diagnostic or therapeutic properties of FAP-targeted applications.

## Figures and Tables

**Table 1:** Results of an investigation of nuclide-labeled FAP-targeting antibodies.

Developer	Name	In Vitro Assessment	Tumor Model	Indication	Phase	Reference
Boehringer Ingelheim	F19 (BIBH-1) and ^131^I-BIBH-1	NA ^1^	Colorectal Cancer Metastatic Cancer	SPECT/CT	Completed	[124] ClinicalTrials.gov Identifier: NCT00005616
Boehringer Ingelheim	^131^I- Sibrotuzumab	Radiochemical purity: ≥95%	Patients of colorectal carcinoma and non-small-cell lung cancer	SPECT	Terminated	[125] ClinicalTrials.gov Identifier: NCT02209727
Eliane Fischer	^177^Lu-ESC11/ESC14	K_d_: 10 nM/ 210 nM	Melanoma xenograft nude mouse	SPECT/CT	Preclinical	[126]
Darpan N. Pandya	[^89^Zr]Zr-Df-Bz-F19	Radiochemical purity: ≥99.5%	U87MG tumor bearing mice	PET/CT	Preclinical	[127]
Hallie M. Hintz	[^89^Zr]Zr- B12-IgG	NA ^1^	Mice bearing intratibial CWR-R1FAP	PET/CT	Preclinical	[128]

^1^ “NA” means “Not Applicable”.

**Table 2:** HPLC analysis of the radiopurity for ^177^Lu-labeled compounds in a formulation buffer containing 100 mg/mL ascorbic acid and 5 mg/mL methionine [216].

Name	Structural Unit	HPLC Retention Time (min)	HPLC Area% Day 0 Post End of Synthesis	HPLC Area% Day 6 Post End of Synthesis
^177^Lu-3BP-3407	Hex-[C(tMeBn(DOTA-PP))-P-P-T-Q-F-C]-D-NH_2_	7.5	95.7	94.0
^177^Lu-3BP-3554	Hex-[C(tMeBn(DOTA-AET))-P-P-T-Q-F-C]-OH	7.6	97.2	95.6

**Table 3:** Structurally akin compounds to FAP-2286. (HEK-FAP Intake (Time) (%ID/g), ++++: 20–25, +++: 15–20, ++: 10–15, +: 6–10, -: <6, C (Cysteine, Cys), D (Aspartic acid, Asp), E (Glutamine Acid), F (Phenylalanine, Phe), G (Glycine, Gly), H (Histidine, His), K (Lysine, Lys), P (Proline, Pro), Q (Glutamine, Gln), R (Arginase, Arg), T (Threonine, Thr), and the dot-circle structure represents, DOTA).

Name	Molecular Formula	Molecular Weight	Structural Unit	Structures (Simple)	HPLC Retention Time (min)	HEK-FAP Intake (Time) in %ID/g
3BP- 3105	C_114_H_169_N_25_O_33_S_2_	2481.171	Hex-[C(3MeBn)-P-P-T-E-F-C]-D-H-F-R-D-Ttds-K(DOTA)-NH_2_	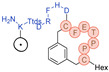	8.9	+ (6 h)
3BP- 3168	C_109_H_161_N_25_O_33_S_2_	2412.110	DOTA-Ttds-Nle-[C-(3MeBn)-P-P-T-E-F-C]-D-H-F-R-D-NH_2_	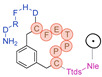	7.9	+ (3 h)
3BP- 3320	C_82_H_124_N_16_O_26_S_2_	1812.841	DOTA-Ttds-Nle-[C-(3MeBn)-P-P-T-E-F-C]-D-NH_2_	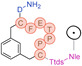	8.6	+ (1 h)
3BP- 3321	C_96_H_143_N_21_O_28_S_2_	2101.993	DOTA-Ttds-Nle-[C-(3MeBn)-P-P-T-E-F-C]-D-Pamb-R-NH_2_	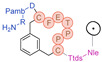	7.4	+ (3 h)
3BP- 3275	C_110_H_165_N_25_O_30_S_2_	2379.820	Hex-[C(3MeBn)-P-P-T-E-F-C]-D-H-Nmf-R-Ttds-K(DOTA)-NH_2_	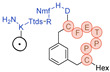	7.3	+ (3 h)
3BP- 3397	C_71_H_106_N_14_O_19_S_2_	1522.720	Hex-[C(3MeBn)-P-P-T-Q-F-C]-Bhk(DOTA)-OH	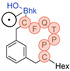	8.3	++ (24 h)
3BP- 3398	C_81_H_124_N_16_O_24_S_2_	1768.842	DOTA-Ttds-Nle-[C-(3MeBn)-P-P-T-Q-F-C]-Bal-OH	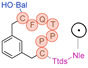	7.3	+ (3 h)
3BP- 3407	C_73_H_108_N_16_O_20_S_2_	1592.737	Hex-[C(tMeBn(DOTA-PP))-P-P-T-Q-F-C]-D-NH_2_	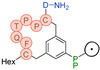	7.3	++ (1/24 h)
3BP- 3554	C_67_H_99_N_13_O_18_S_3_	1469.640	Hex-[C(tMeBn(DOTA-AET))-P-P-T-Q-F-C]-OH	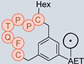	7.5	++++ (6 h)
3BP- 3652	C_67_H_100_N_14_O_17_S_3_	1468.667	Hex-[C(tMeBn(DOTA-AET))-P-Q-F-C]-NH_2_	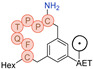	7.3	+++ (1 h)
3BP- 3654	C_66_H_99_N_13_O_16_S_3_	1425.661	Hex-[C(tMeBn(DOTA-AET))-P-P-T-Q-F-AET]	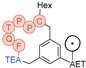	7.8	+ (1 h)
3BP- 3656	C_69_H_102_N_14_O_19_S_3_	1526.665	Hex-[C(tMeBn(DOTA-AET))-P-P-T-Q-F-C]-G-OH	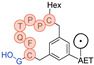	7.3	-
3BP- 3659	C_70_H_104_N_14_O_19_S_3_	1540.682	Hex-[C(tMeBn(DOTA-AET))-P-P-T-Q-F-C]-Nmg-OH	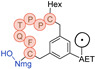	7.3	-
3BP- 3678	C_65_H_97_N_13_O_18_S_3_	1443.624	Hex-[C(tMeBn(DOTA-AET))-Nmg-P-T-G-Q-C]-OH	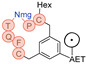	7.4	+ (1 h)
3BP- 3692	C_72_H_108_N_16_O_21_S_3_	1628.706	Pentyl-SO_2_-[C(tMeBn(DOTA-PP))-P-P-T-Q-F-C]-D-NH_2_	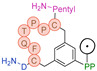	7.8	+ (3 h)
3BP- 3767	C_67_H_101_N_13_O_17_S_3_	1455.666	Hex-[C(tMeBn(DOTA-AET))-P-P-T-Q-F-Cysol]	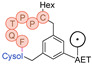	7.4	++ (1 h)

## Data Availability

Data can be found within the article.

## Abbreviations

CAF	Cancer-associated Fibroblast
CT	Computerized Tomography
DPP	Dipeptidyl Peptidase
FAP	Fibroblast Activation Protein
FAPI	FAP Inhibitor
FDA	Food and Drug Administration
IC_50_	Half Maximal Inhibitory Concentration
ID/g	Injected Dose/Gram
MAb	Monoclonal Antibody
mCRPC	Metastatic Castration-resistant Prostate Cancer
MIP	Maximum Intensity Projection
NIR	Near-infrared
PET	Positron Emission Tomography
PK	Pharmacokinetic
SPECT	Single Photon Emission Computed Tomography
TME	Tumor Microenvironment
T/K	Tumor-to-kidney Ratio
